# Employers’ utilization of and collaboration with occupational health services in preventive occupational health and safety management

**DOI:** 10.5271/sjweh.4269

**Published:** 2026-05-01

**Authors:** Magnus Akerstrom, Jens Wahlström, Cathrine Reineholm, Ingibjörg H Jonsdottir

**Affiliations:** 1 Institute of Stress Medicine, Region Västra Götaland, Gothenburg, Sweden.; 2 School of Public Health and Community Medicine, Institute of Medicine, Sahlgrenska Academy at University of Gothenburg, Gothenburg, Sweden.; 3 Department of Epidemiology and Global Health, Umeå University, Umeå, Sweden.; 4 Department of Behavioural Sciences and Learning, Linköping University, Linköping, Sweden.

**Keywords:** OSH, performance, prevention

## Abstract

**Objectives:**

Occupational health services (OHS) are an important resource within employers’ preventive occupational health and safety management (OHSM). The aims of this study were to investigate employers’ utilization of OHS in preventive OHSM and identify decisive contextual, structural and/or processual conditions in ensuring successful collaboration with OHS in preventive OHSM.

**Methods:**

A total of 122 organizations within the Swedish welfare sector (education, social services and healthcare), of which 112 had access to OHS, responded to a quantitative survey on the organization and management of their preventive OHSM. Responses were investigated using both conventional descriptive analysis and configurational analysis.

**Results:**

Only a third of the participating organizations utilized OHS to a high or very high degree within their preventive OHSM. Factors for successful collaboration with OHS within preventive OHSM included having a contract that made it possible to use OHS and having established routines for involving OHS in the early phases of preventive measures, especially when managing external demands and complex challenges.

**Conclusions:**

The utilization of OHS within preventive OHSM is limited, and increased utilization may improve the ability of employers to successfully implement OHSM. The successful organization of OHSM practices and the OHS contract were key factors in a successful collaboration with OHS. Working together in preventive OHSM may also strengthen social capital for all stakeholders, which could further enhance collaboration.

Employers in Europe, including Sweden, have been obliged by law to provide a safe work environment for more than three decades, and occupational health and safety management (OHSM) must be performed systematically to prevent and manage work-related health problems (1–3). Nevertheless, work-related mental health problems remain a global challenge, with many workplaces struggling with increasing rates of sickness absence and high employee turnover, particularly in the public sector (4–6). There is therefore an urgent need for increased knowledge about the factors that hinder or support a successful OHSM.

Employers’ lack of knowledge in preventive OHSM has been identified as one of several factors contributing to the inconsistent success of OHSM initiatives (7–9). Occupational health services (OHS) are external resources that could potentially be used to improve employers’ competence in OHSM and occupational health (7, 10, 11). In Sweden, state subsidies for OHS were abolished in the early 1990s, which meant that employers had to bear the entire cost themselves (12). OHS then began to function more as market players, where services were adapted to employer demand. Since then, OHS has been shown to be an underutilized resource within preventive OHSM (10, 13–15).

To increase the utilization of OHS, a successful collaboration between employers and their OHS has been shown to be important (10, 16, 17). To date, there is some knowledge about factors that influence the success of the collaboration between employers and their OHS, such as the OHSM structure, the content of contracts with OHS, the role OHS is given in organizations, the social capital within OHS, long-term relationships and dialogue that targets different organizational levels within workplaces (10, 14, 15, 17). However, most of this research has relied on qualitative study designs with a limited number of organizations, which calls for quantitative studies including a wider range of organizations.

Thus, by employing a novel approach with a mixed-methods design, this study had three aims: to (i) investigate employers’ utilization of OHS in preventive OHSM; (ii) identify difference-making contextual, structural and/or processual conditions for having respectively not having a successful collaboration with OHS; and (iii) investigate the association between successful collaboration with OHS and preventive OHSM practices.

## Methods

### Study design and setting

This cross-sectional study employed a mixed-methods approach for a more comprehensive and nuanced understanding by combining conventional statistical analysis and the novel configurational analysis (18). Configurational comparative methods are based on an alternative theoretical foundation, set theory, to what is conventionally used within this field. Whereas statistical analysis is variable-oriented and relies on correlational analysis to make comparisons across cases, configurational comparative methods are based on set theory, are case oriented and rely on Boolean algebra to make comparisons between cases, which has been suggested to better embrace the complexity in studies of OHSM (19).

This study is a part of a larger project within the Swedish welfare sector (healthcare, social services, education, public administration and workplaces providing services connected to welfare). The Swedish welfare sector consists mainly of public workplaces within 290 municipalities, which are responsible for services such as education, social services and elder care, and 21 regions, which are mainly responsible for services such as healthcare and regional development. A number of privately owned businesses operating within the publicly funded welfare sector were also included in this study.

### Data collection and participating workplaces

To collect organizational data, rather than the perception from individuals within the organization, the Hybrid response process model (20) was used. In this data collection strategy, invited organizations themselves do assess whether they are eligible to participate or not. Criteria for inclusion were operating within the welfare sector, conducting a preventive OHSM, and being able to nominate one or more employer representatives, depending on their organization of the preventive OHSM, that could respond on behalf of the organization. Consequently, the exclusion criteria were not operating within the welfare sector, not conducting a preventive OHSM (eg, being self-employed) and not being able to nominate a representative that could respond to the survey on behalf of the organization. An electronic survey was distributed to all municipalities, regions and registered privately owned businesses, using public records and registers from the Swedish Association of Local Authorities and Regions, the Health and Social Care Inspectorate and the Swedish National Agency for Education together with information on how to respond to the survey. Social media (Facebook and LinkedIn), the research group’s external webpage, unions and other professional networks, and external collaborations was also used to disseminate information about the study, as previously described (21).

As there were no existing questionnaires to determine how employers initiate, design and implement preventive occupational health interventions within their preventive OHSM available, a quantitative questionnaire was developed and tested prior to this study. The development and testing of the survey has previously been reported (21). Briefly, the questionnaire was developed based on interviews with key actors, experts and intended respondents. The final questionnaire was tested using qualitative and quantitative analyses of open-ended and multi-choice items to assess the response distribution and the content validity. In addition, the interrater reliability was assessed as the employer-employee correspondence from 32 matched pairs from the same workplace. Results from the testing of the questionnaire indicated a high content validity with a satisfactory response distribution and very small proportion of missing data on individual items. Also, the interrater reliability was overall assessed as high (>60%), but the employer representatives were generally found to possess a higher information power based on the higher proportion of the response option “I don’t know” for employee representatives on items related to the organization of the OHS within the preventive OHSM.

A total of 126 organizations responded to the invitation to participate in the study and provided organizational responses to the survey. The data collection strategy did not allow calculation of a response rate, as there was no information on whether non-responding organizations considered themselves eligible for participation. However, assuming that all regions and municipalities were eligible, responses were received from 11 of Sweden’s 21 regions and 70 of its 290 municipalities. Overall, the employer representatives were most often (72%) from human resources (HR), especially in the municipalities and the regional councils, but they also included managers (13%), senior managers (7%) and work environment coordinators (7%) (21). Four survey responses were excluded because the organizations were not part of the welfare sector (N=2), the responses were incomplete (<50% of items answered, N=1), or they were duplicate responses from the same workplace (N=1), resulting in 122 participating organizations of which 112 had access to OHS. With exception for the comparisons of characteristics between organizations with and without access to OHS, presented in the first paragraph of the results, only participating organizations with access to OHS was included in the analyses (table 1).

**Table 1:** Descriptives and the workplaces organization of their collaboration with their occupational health services (OHS) for the 112 participating organisations with access to OHS. [OHSM=occupational health and safety management]

		Total ^a^		Type of OHS provider		Difference between type of OHS provider (P-value)
		N (%)		Internal N (%)	External N (%)	
Organisations with access to OHS		112 (100)		19 (17)	93 (83)	
Sector						<0.001
	Municipality	74 (66)		5 (26)	69 (74)	
	Region	22 (20)		13 (68)	9 (10)	
	Private	16 (14)		1 (5)	15 (16)	
Number of employees						0.4
	<20	5 (5)		1 (5)	4 (4)	
	20–49	9 (8)		0 (0)	9 (10)	
	50–249	11 (10)		1 (5)	10 (11)	
	>250	85 (77)		17 (89)	68 (75)	
Type of contract with OHS						<0.001
	Subscription with full-service package	14 (13)		7 (37)	7 (8)	
	Subscription with basic services and ordering	26 (24)		9 (47)	17 (19)	
	Ordering with basic fee	16 (15)		0 (0)	16 (18)	
	Ordering without basic fee	43 (39)		2 (11)	41 (45)	
	Unknown	11 (10)		1 (5)	10 (11)	
How preventive measures are ordered						0.6
	We have not ordered preventive measures	2 (2)		0 (0)	2 (2)	
	Standard pre-packed measures are ordered	2 (2)		1 (5)	1 (1)	
	Standard pre-packed measures, somewhat tailored measures are ordered	25 (22)		4 (21)	21 (23)	
	Measures tailored for our needs are ordered	80 (71)		13 (68)	67 (72)	
	Unknown	3 (3)		1 (5)	2 (2)	
Organisation of collaboration with OHS						0.09
	Meetings only in connection to ordering of measures	33 (29)		5 (26)	28 (30)	
	Regular meetings to share information apart from specific measures	51 (46)		6 (32)	45 (48)	
	Close collaboration on different levels in the organisation	24 (21)		8 (42)	16 (17)	
	Unknown	4 (4)		0 (0)	4 (4)	
Successful collaboration with occupational health services within the preventive OHSM						0.7
	Strongly disagree	4 (4)		0 (0)	4 (4)	
	Somewhat disagree	7 (6)		1 (5)	6 (7)	
	Neither unsuccessful nor successful	48 (43)		4 (21)	44 (48)	
	Somewhat agree	45 (41)		11 (58)	34 (37)	
	Strongly agree	7 (6)		3 (16)	4 (4)	

^a^ The participating organizations nominated one or more representatives depending on their organization of preventive OHSM resulting in multiple responses (covering different parts of the organisation) for some of the larger regions and municipalities. In total, 11 of the 21 regions in Sweden and 70 of the 290 municipalities in Sweden were represented in the data.

### Measures

The questionnaire contained 86 items related to contextual factors (eg, size and type of operations differentiating one workplace from another), structural factors (eg, how the OHSM and collaboration with OHS are organized and access to resources), processual factors (eg, how OHS are used and how interventions within the OHSM are designed and implemented) and outcomes such as the satisfaction with the collaboration with OHS and preventive OHSM practices (eg, proportion of work environment measures that are preventive or on an organizational level within the OHSM and the extent to which the OHSM was assessed to prevent work-related ill-health or promote health). Multiple-choice responses were used for contextual items to describe structural aspects of how OHSM and the collaboration with OHS have been organized. Five Likert-scale response alternatives, ranging from “to a very low degree” to “to a very high degree”, were used to describe the utilization of OHS, how interventions within the OHSM are designed and implemented and the outcomes of this work.

All questionnaire items examining the organization of collaboration with OHS and how OHS are utilized when employers initiate, design and implement preventive occupational health interventions within their preventive OHSM were used in the statistical analyses. In the configurational analyses, all items were used as described below. The main outcome in this study was measured with the item “Overall, we have a successful collaboration with the occupational health services in the preventive/promotive OHSM” with five Likert-scale response alternatives ranging from “strongly disagree” to “strongly agree”. These responses were dichotomized into *successful collaboration* (“somewhat agree” and “strongly agree”) and *not successful collaboration* (“neither agree nor disagree”, “somewhat disagree” and “strongly disagree”). As described above, a high content validity and a satisfactory response distribution was found for the questionnaire, including the main outcome, and the interrater reliability for this specific item was high (75%) (21). For the third aim of the study, items exploring the proportion of preventive or organizational measures within OHSM and the perception of successful preventive OHSM when it comes to preventing illness and accidents or promoting health among employees were used as outcomes.

### Statistical analyses

The employers’ utilization of OHS in preventive OHSM (the first aim) was investigated using descriptive statistics and included an investigation of differences between sectors. Due to the somewhat limited sample size, associations between successful collaboration with OHS and the utilization of OHS and OHSM practices (aims i and iii) were investigated by examining differences between workplaces with and without successful collaboration with OHS. Differences between groups were investigated using the chi-square test (for binomial variables) or Kruskal-Wallis test (for non-binomial variables), SAS version 9.4; SAS Institute, Cary, NC, USA. P≤0.05 were considered statistically significant.

### Configurational analysis

To identify difference-making conditions (ie, a bundle of specific factors) influencing the presence or absence of successful collaboration with OHS (aim ii), configurational analyses were conducted. Coincidence analysis (CNA) was used with the R package “cna”, [R Statistical Software v.4.3.2, R Core Team, 2023; RStudio v.2023.12.1+402; R package “cna” v.3.5.4 (22)]. CNA is a case-based, mathematical approach to identify key conditions that uniquely distinguish a group of cases with an outcome of interest from another group without that outcome. In this study, CNA was selected for its ability to model conjuncts (where several conditions must be present together to produce the outcome) and disjuncts (where multiple pathways lead to the same outcome). The method also allows separate models to be derived for positive models (solutions where the outcome is present) and negative models (solutions where the outcome is absent) (23–26). The analysis was performed in three steps: factor calibration, data reduction and model development (25). Data reduction and model development were conducted separately for the presence and absence of successful collaboration with OHS.

### Factor calibration

Factors originating from items using Likert-scale alternatives were dichotomized to create a crisp data set (ie, a data set containing only binary factors) for the model development step. Dichotomization was used to decrease the complexity of the data in relation to the somewhat limited study sample. To facilitate the interpretation of the result, Likert-scale alternatives were generally dichotomized as being present (agree to a high or a very high degree) or not present (neither agree nor disagree, somewhat disagree and strongly disagree). Multiple-choice response alternatives from the questionnaire were used in the initial data reduction step, and, if identified as candidate factors, dichotomized based on the mathematical output (23) prior to the model develop step. The included factors and their calibration can be found in the supplementary material, www.sjweh.fi/article/4269, appendix 1.

### Data reduction

Due to the large number of included factors, a multi-step inductive bottom-up approach for data reduction was used. This was in line with the explorative aim of this study, as there were no compelling a priori reasons to select certain factors over others. The data reduction was made using the “minimally sufficient conditions” (msc) routine within the R package “cna” which have been previously described (27, 28). A more thoroughly description of the application of how the msc routine was applied in this study, and the criteria for factor selection used, can be found in supplementary appendix 2.

### Model development

In the model development phase, the model-building function for crisp data sets within the “cna” package in R was used. The goal was to produce a model that explained at least two out of three (67%) workplaces with the outcome (coverage), yielded the outcome at least 80% of the time the solution appeared anywhere in the data set (consistency), was aligned with theory and logic, and contained no model ambiguity (27, 29). At this step, any cases with missing values were dropped, which is standard procedure in configurational analysis, where important conditions are prioritized over additional cases. This process ultimately resulted in 109 cases for the positive model (ie, presence of successful collaboration) and all 112 cases for the negative model (ie, absence of successful collaboration).

## Results

In total, 122 employer representatives within the Swedish welfare sector (education, social services and healthcare) responded to the survey and provided information on the utilization, organization and outcomes of their preventive OHSM, including their collaboration with OHS. Most of the 122 participating organizations (92%, N=112) had access to OHS. Organizations without access to OHS were more likely to belong to the private sector (P<0.001) and to have <50 employees (P<0.001). Among organizations with access to OHS, 19 (17%) had an internal OHS and 93 (83%) used external OHS. It was more common to have internal OHS (P<0.001) within the regional sector compared to the municipal and private sector. In addition, organizations with an internal OHS were more likely to have contracts that included basic services and other services, rather than ordering all services on demand, which was more common for organizations with an external OHS (P<0.001). No differences were observed between organizations with internal and external OHS in how preventive measures were ordered or in the level of satisfaction with their collaboration with OHS (see table 1).

### Utilization of OHS in preventive OHSM and its association with a successful collaboration

Half of the participating organizations with access to OHS used the services to identify the need for preventive measures (54%) to a high or very high degree. About a third (29–35%) of the 112 organizations with access to OHS reported they used services to a high or very high degree in designing, making decisions, and implementing measures within the preventive OHSM. Meanwhile, about a third of the organizations reported doing so to a low or a very low degree (27–38%).

An association was observed between successful collaboration with OHS and the form of collaboration (eg, meetings when needed or more regular meetings and collaborations) and whether OHS was used in preventive OHSM, with an increasing degree of satisfaction with closer collaboration and greater use of OHS in preventive OHSM (P=0.005; P<0.001, respectively). No association was seen between the degree of satisfaction with the collaboration and the type of OHS (internal or external; P=0.7), the type of contract (P=0.2) or process of ordering services (P=0.3).

### Difference-making conditions for the presence of a successful collaboration with OHS within preventive OHSM

The exploratory data reduction step identified eight contextual, structural and processual candidate factors that were directly linked to successful collaboration with OHS. These eight candidate factors were selected for use in the model development phase (see supplementary appendix 1) and included the nature of work environment challenges, whether senior management considered it important to invest in competence development, how preventive OHSM and collaboration with OHS were organized and the degree to which OHS were used in decisions and the design of preventive interventions.

**Figure 1:**
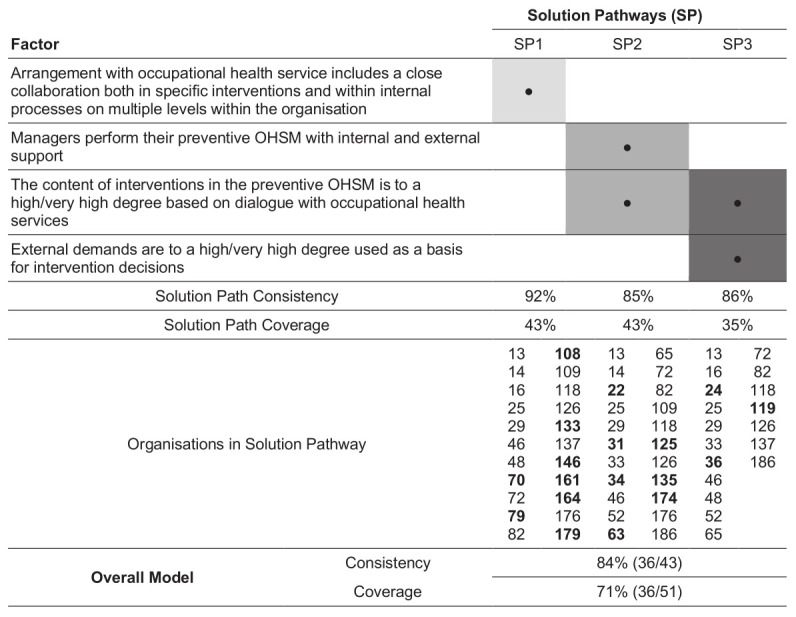
Final model for the positive outcome (having a success collaboration with occupational health services (OHS) within the preventive occupational health and safety management (OHSM). The figure presents the individual factors included in each of the final three-pathway model, together with consistency, coverage and organisations covered both overall and the respective solution pathway. Organizations listed in bold are only explained by one solution pathway. After excluding organisations with missing data, this model included 109 of the 112 initial organizations.

The configurational analysis yielded five potential solutions at chosen levels of consistency and coverage (ie, ≥80% and 67%, respectively), but only one solution was aligned with theory and logic. This solution consisted of a three-pathway model comprising four structural and processual conditions (see figure 1). The model accounted for 36 of the 51 organizations with successful collaboration with OHS, resulting in an overall coverage of 71%. Seven organizations without a successful collaboration were identified by the model, resulting in an overall consistency of 84% (see supplementary appendix 3).

The three pathways consisted of organizations with (i) OHS contracts that included close collaboration both in specific interventions and in everyday processes of organizational development; (ii) established routines where managers performed preventive OHSM with internal and external support, and where the content of interventions was based to a high or very high degree on dialogue with OHS; or (iii) external demands which were used to a high or very high degree as a basis for decisions on implementing interventions, and where the content of interventions was based to a high or very high degree on dialogue with OHS (see figure 1).

**Figure 2:**
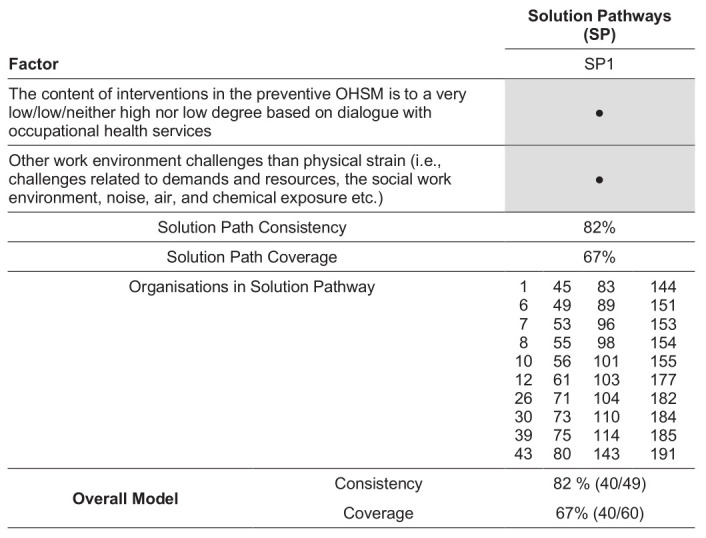
Final model for the negative outcome (not having a successful collaboration with occupational health services (OHS) within the preventive occupational health and safety management (OHSM) for the 112 participating organizations with an OHS contract. The figure presents the individual factors included in the final one-pathway model, together with consistency, coverage and organisations covered by this solution pathway.

### Difference-making conditions for the absence of a successful collaboration with OHS within preventive OHSM

The exploratory data reduction step identified five contextual, structural and processual candidate factors that were directly linked to the absence of successful collaboration with OHS. These candidate factors were selected for use in the model development phase (supplementary appendix 1) and included the nature of work environment challenges, how preventive OHSM and collaboration with the OHS were organized and to what degree OHS were utilized in decision-making and the design of preventive interventions.

The configurational analysis only yielded one solution at the chosen levels of consistency and coverage. This was a single-pathway model comprised of one structural and one processual condition (see figure 2). The model accounted for 40 of the 60 organizations without a successful collaboration with OHS, resulting in an overall coverage of 67%. Nine organizations were identified by the model with a successful collaboration, resulting in an overall consistency of 82% (see supplementary appendix 4).

The identified pathway consisted of organizations that based the content of interventions on dialogue with OHS to a very low or low degree, or neither high nor low degree, and where work environment challenges other than physical strain (ie, challenges related to demands and resources; the social work environment; noise, air, and chemical exposure; etc.) were present (see figure 2).

### Association between successful collaboration with OHS and preventive OHSM practices

**Figure 3:**
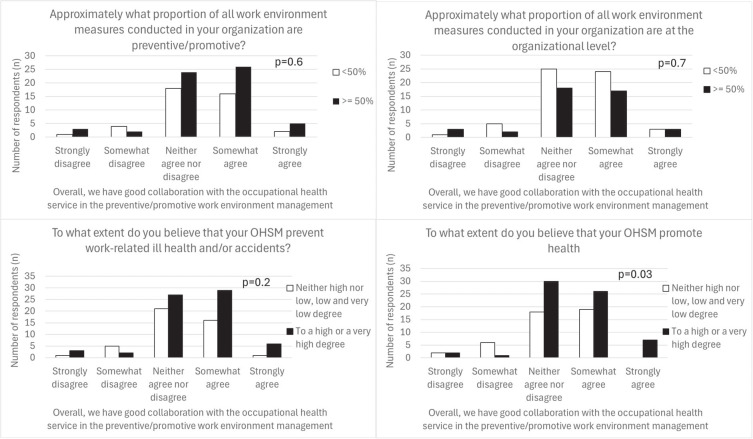
Association between the employers perception on the collaboration with their occupational health services (OHS) and their self-assessed proportion of preventive/promotive and organizational measures within the occupational health and safety management (OHSM), as well as their perceived success of OHSM in terms of preventing ill health and accidents and promote employee health, respectively.

There were no significant associations found between perceived successful collaboration with OHS and the proportion of preventive or organizational measures within OHSM (P=0.6 and P=0.7, respectively), or the perception that successful OHSM prevents illness and accidents among employees (P=0.2). However, there was a small but significant association between the level of satisfaction with the collaboration and the perception that successful OHSM promotes the health of employees (P=0.03) (see figure 3).

## Discussion

This study aimed to investigate employers’ utilization of OHS in preventive OHSM and to identify contextual, structural and/or processual factors that promote successful collaboration with OHS in preventive OHSM. The results showed that the utilization of OHS was limited, and only half of the 112 participating organizations experienced a successful collaboration with OHS within preventive OHSM. Factors associated with a successful collaboration included having an OHS contract that allowed for close collaboration in both specific interventions and in everyday processes of organizational development, and having established routines and a culture of involving OHS in preventive OHSM. The results further indicate that the latter may be especially important when facing external demands and complex challenges.

Previous research shows that employers’ utilization of OHS in preventive OHSM is limited (10, 13–15). The results of the present study confirm this, showing that only half of the participating organizations utilized OHS to a high degree when the need for improvement was identified. Furthermore, only one in three organizations utilized OHS to a high degree when designing and making decisions on implementing measures within preventive OHSM. The low utilization of OHS may not be problematic for organizations as long as they have access to other sources of support. However, previous studies have identified employers’ lack of knowledge in preventive OHSM and occupational health as one of several factors contributing to the inconsistent success of OHSM initiatives (7–9). Employers have also reported a lack of support in implementing preventive OHSM from internal support functions such as HR, which operate on a more structural level within OHSM rather than offering practical support in preventive OHSM (30). This shows that OHS may be an underutilized resource for organizations within the Swedish welfare sector.

The results also indicate that the underutilization of OHS may be negatively associated with the perceived success of the collaboration between employers and OHS within preventive OHSM, and only half of the participating organizations reported a successful collaboration, which may further hinder utilization. This aligns with earlier findings highlighting the importance of networking and collaboration with OHS to strengthen social capital (eg, trust, norms, social support, information channels and social credentials) (12, 17, 31). This may be especially important in Sweden, where OHS is based on voluntary contracts between employers and OHS providers operating in a market-driven environment (12, 17).

By employing configurational analyses to identify difference-making factors for having a successful collaboration with OHS, instead of supporting and hindering factors as has been investigated in previous research, this study provides new insights into potential measures that may be used to increase collaboration in preventive OHSM. Interestingly, these factors (eg, contractual relationship with OHS and established routines and practices within OHSM) all originated from the organizational level. This indicates that senior management play an important role in increasing utilization of and collaboration with OHS in preventive OHSM. Contractual relationships and practices have previously been identified as both supporting and hindering factors in OHSM (10, 14, 15). However, this study highlights the importance of these areas for successful collaboration with the OHS within OHSM and identifies potential areas that plausibly could be prioritize when navigating the large number of potentially supporting and hindering factors. It also highlights the importance of strong social capital among all stakeholders for increased collaboration with the OHS within preventive OHSM.

Furthermore, the findings also indicate that the greatest need for collaboration with OHS arises when facing external demands and complex challenges, which has also been observed in previous studies (7–9). This shows that OHS need to develop skills to meet the needs of their customers.

As for all case-oriented methods, generalizing the findings to a wider context must be done with caution due to both the method selection and the limited representativeness of the included organizations. While cross-sectional studies are valuable for describing prevalence and associations at a specific point in time, they do not allow for causal inference or assessment of temporal relationships between exposure and outcome. Employing a quantitative approach within the public welfare sector (ie, healthcare, social services, education, public administration and workplaces providing services connected to welfare) enables access to a wider range of organizations compared to most previous studies using qualitative design with a limited number of organizations. In addition, configurational analyses have been shown to generate valid results even for studies with small study samples (24, 25). Despite the somewhat small study sample, 11 of the 21 regions in Sweden and 70 of the 290 municipalities in Sweden were represented with one or more responses.

Lastly, in contrast to earlier studies (10, 16, 17), this study failed to show an overall association between successful collaboration and the outcomes of preventive OHSM, except for the perception that OHSM promotes health. However, this may have been affected by the limited study sample and outcome measures or the overall low utilization of OHS among organizations surveyed in the present study. Furthermore, the presence of an association between a successful collaboration with OHS and the perception that OHSM promotes health is interesting since this has been shown to be a major challenge for employers suggesting an increased need for support (32). Therefore, more knowledge is needed to understand the association between utilization, perceived collaboration and the performance of promotive and preventive OHSM.

### Strengths and limitations

A strength in this study is the use of a mixed-methods approach, enabling us to gain a deeper understanding of factors for successful collaboration with OHS within preventive OHSM. In addition, the configurational method – coincidence analysis – has been shown to generate valid results for studies with a wide range in the number of participants, including studies with small numbers of participants (24, 25).

This study had a number of limitations. The limited sample size and the cross-sectional study design limits the interpretation of the findings and the possibility to adjust for potential confounding variables in the statistical analyses. Even though the vast majority of the participating organizations had access to OHS, the actual use of OHS in the preventive OHSM was somewhat low, which has also been noted in previous studies (10, 13–15). This limits the generalization to other high utilization contexts. Although using a quantitative survey enabled access to a larger study population, it only allowed examination of the extent of collaboration rather than its quality. Furthermore, the outcome measures in this study were limited to self-assessments on a limited aspects related to collaboration with OHS and performance of the OHSM. Including a wider range of outcome measures, including objective measures as sickness absence data, turnover rates etc, would have strengthen the results further.

### Concluding remarks

OHS are an underutilized resource for most organizations within the Swedish welfare sector, despite the challenges employers face in preventive OHSM. Consequently, only half of the organizations reported a successful collaboration with OHS in preventive OHSM. The findings of this study indicate that sufficient organizational preconditions need to be in place, both in terms of contractual options to include OHS in OHSM and established routines where OHS are utilized in practice, to increase collaboration with OHS. Our findings also indicate that this may be especially important when facing external demands and complex challenges. Collaboration with OHS in preventive OHSM may also strengthen the social capital for all stakeholders, which could increase the collaboration further.

## Supplementary material

Supplementary material

## Data Availability

The data sets generated and/or analyzed during the current study are available from the corresponding author on reasonable request.
