# Supplementary material for: Employers’ utilization of and collaboration with occupational health services in preventive occupational health and safety management
Source: Scand J Work Environ Health. 2026 Apr 30;52(3):333–42. doi: 10.5271/sjweh.4269 (PMC13189005; doi:10.5271/sjweh.4269)
Supplement: Supplementary material [file SJWEH-52-333-S001.pdf]

Employers' utilization of and collaboration with occupational health services in preventive occupational health and safety management[1]

by Magnus Akerstrom, PhD[2] *Jens Wahlström, PhD, Cathrine Reineholm, PhD, Ingibjörg H Jonsdottir, PhD*

1. Supplementary material
2. Correspondence to: Magnus Akerstrom, Institute of Stress Medicine, Västra Götaland Region, Gothenburg, Sweden, Carl Skottbergs gata 22B, 413 19 Gothenburg, Sweden. [E-mail: magnus.akerstrom@vgregion.se]

Appendix 1

Factors used in the CNA analyses

Factor	Calibration	Basis for calibration
**Contextual factors (n=14)**		
Collective agreement	0 = no<br>1 = yes	a
Work environment challenges related to demands and resources		a
Work environment challenges: threats and violence		a
Work environment challenges: harassment and bullying		a
Work environment challenges: conflicts		a
Work environment challenges: physical strain [f, g]		a
Work environment challenges: noise, air, and chemical exposure		a
Work environment challenges: accidents or safety risks		a
Work environment challenges: other		a
Challenges in social work environment		a
Challenges in physical work environment [d]		a
In how many categories there are challenges (job demand, social, physical)	0 = challenges in 0 or 1 category<br>1 = challenges in 2 or 3 categories	a
Organisation size	1 = small: up to 19 employees<br>2 = medium-small: 20-49 employees<br>3 = medium: 50-249 employees<br>4 = large: 250 or more employees	b
Sector	1= Private<br>2 = Municipality	a

	3 = Region<br>4 = State	
**Structural factors (n=27)**		
Organisation of the systematic occupational health and safety management [d, e]	1= Manager without support<br>2 = Manager with support from developed routines and work methods<br>3 = Manager with support from internal resources<br>4 = Manager with support from internal and external resources<br>5 = Manager has delegated the responsibility	b
Use of measurable goals related to work environment regularly monitored	0 = No, partially, do not know<br>1 = Yes	c
Use of goals related to organizational and social work environment		c
Deficiencies in the work environment reported by employees used as a basis for intervention decisions	0 = low/very low/neither low nor high extent, do not know<br>1 = High/very high extent	c
Analysis of employee data used as a basis for intervention decisions		c
Analysis of operational data used as a basis for intervention decisions		c
HR analysis used as a basis for intervention decisions		c
Expected profitability used as a basis for intervention decisions		c
Dialogue with safety delegates/union representatives used as a basis for intervention decisions [d]		c
Dialogue with occupational health service used as a basis for intervention decisions [f]		c
Dialogue with internal resources used as a basis for intervention decisions		c
Internal improvement processes used as a basis for intervention decisions		c
External demands used as a basis for intervention decisions [d,e]		c
Plan for how the intervention will be evaluated		c
Budget enabling investment in improved work environment		c
Budget enabling investment in competitive salaries		c
Budget enabling investment in competence development		c
Budget enabling investment in employer brand		c

Budget enabling investment in environmental sustainability		c
Sufficient resources to analyse underlying causes of work environment challenges	0 = somewhat/strongly disagree, neither agree nor disagree, do not know<br>1 = somewhat/strongly agree	c
Sufficient knowledge to select relevant interventions [d]		c
Access to methods and routines to select relevant interventions		c
How the budget for interventions is organized	0 = cost centralized<br>1 = cost charged to manager's budget	a
Type of occupational health services	1 = Internal OHS<br>2 = External OHS	a
Agreement with occupational health services	1= Subscription agreement with full-service package<br>2= Subscription agreement with basic services and ordering<br>3 = Ordering agreement with basic fee<br>4 = Ordering agreement without basic fee<br>9 = Do not know	b
Ordering of occupational health services	0 = Do not order interventions<br>1 = Ordering based on pre-packaged interventions<br>2 = Ordering based on pre-packaged interventions, somewhat tailored<br>3 = Ordering always tailored<br>9 = Do not know	b
Access to well-functioning cooperation agreements	0 = low/very low/neither low nor high extent, no cooperation agreement<br>1 = High/very high extent	c
**Processual factors (n=46)**		
Top management involved when the need for intervention is identified	0 = Function does not exist, low/very low/neither low nor high extent<br>1 = High/very high extent	c
Manager with employee responsibility involved when the need for intervention is identified [f]		c
HR involved when the need for intervention is identified		c
Safety delegate involved when the need for intervention is identified		c
Union representative involved when the need for intervention is identified		c
Occupational health service involved when the need for intervention is identified		c

Employees involved when the need for intervention is identified		c
Content of interventions based on dialogue with occupational health services [d, e. f, g]		c
Content of interventions based on dialogue with internal expert resources		c
Content of interventions based on dialogue with safety delegates/union representatives		c
Content of interventions based on dialogue with employees		c
Content of interventions based on evidence-based methods		c
Content of interventions based on knowledge of interventions conducted in other organizations		c
Content of interventions based on the organization's conditions and situation		c
Content of interventions based on existing work methods and tools		c
Content of interventions based on previous experiences from the organization		c
Use of results from previous interventions to develop a long-term strategy		c
Leadership considers it important to invest in improved work environment	0 = low/very low/neither low nor high extent, do not know<br>1 = High/very high extent	c
Leadership considers it important to invest in salaries		c
Leadership considers it important to invest in competence development [d]		c
Leadership considers it important to invest in employer brand		c
Leadership considers it important to invest in environmental sustainability		c
Estimation of total cost when interventions are to be implemented		c
Return on investment considered when interventions are implemented		c
Cost savings considered when interventions are implemented		c
Cost-effectiveness is considered when interventions are implemented		c
Improved productivity is considered when interventions are implemented		c
Improved work environment is considered when interventions are implemented		c
Improved employee health is considered when interventions are implemented		c

Would like return on investment to be considered more when interventions are implemented	0 = less/much less/neither less nor more, do not know<br>1 = More/much more	c
Would like cost savings to be considered more when interventions are implemented		c
Would like cost-effectiveness to be considered more when interventions are implemented		c
Would like productivity to be considered more when interventions are implemented		c
Would like improvement of work environment to be considered more when interventions are implemented		c
Would like improvement of employee health to be considered more when interventions are implemented		c
The content of interventions is anchored with managers at several levels	0 = somewhat/strongly disagree, neither agree nor disagree, do not know<br>1 = somewhat/strongly agree	c
The content of interventions is anchored with managers with employee responsibility		c
The content of interventions is anchored with internal support functions		c
The content of interventions is anchored with employees		c
The content of interventions is anchored with union representatives		c
The content of interventions is anchored with occupational health service		c
The content of interventions is anchored with safety delegates		c
Arrangement with occupational health service [d, e, f]	1 = meet as needed<br>2 = regular meetings<br>3 = close collaboration	a
Proportion of preventive/promotional interventions implemented at the workplace	0 = <25%, 26-50%, do not know<br>1 = 51-75%, 76-100%	c
Proportion of organizational-level interventions implemented at the workplace		c
Extent of preventive/promotional interventions in the last three years	0 = no or occasional preventive interventions<br>1 = several preventive interventions<br>2 = continuous improvement	c
**Outcome factors (n=1)**		
Successful collaboration with occupational health services	0 = somewhat/strongly disagree, neither agree nor disagree<br>1 = somewhat/strongly agree	c

[a]Binary or multi-value category in the survey, [b]Theoretical assumptions, [c]Natural gaps in data, [d]Remaining after the data reduction for the positive model, [e]In the final positive model, [f]Remaining after the data reduction for the negative model, [g]In the final negative model

Appendix 2

Methods

Data reduction

Due to the large number of included factors, a multi-step inductive bottom-up approach for data reduction was used. This was in line with the explorative aim of this study, as there were no compelling a priori reasons to select certain factors over others. The "minimally sufficient conditions" (msc) routine within the R package "cna" was used. In this process, we exhaustively considered all possible combinations of one-, two- and three-condition configurations in the data to identify specific configurations of conditions strongly linked to the outcome of interest (i.e., successful collaboration with OHS). This step was performed separately for the presence and absence of successful collaboration with OHS. Each identified configuration was assessed against a prespecified consistency threshold (i.e., model reliability calculated as the number of cases covered by the model where the outcome is present, divided by the total number of cases covered by the model). The "msc" routine was run multiple times at different prespecified consistency thresholds (95%, 90%, 85%, 80% and 75%) and configurations that met the specific threshold were retained and organised in a "condition table" where rows represent individual configurations and columns represent values for the outcome, conditions, consistency, coverage (i.e., explanatory breadth calculated as the number of cases covered by the model where the outcome is present, divided by the total number of cases with the outcome present) and complexity (i.e., one-, two- or three-condition configurations). This condition table was then reviewed to identify candidate conditions for the model development phase that met the following four criteria: 1) top coverage score within configurations of the same complexity level, 2) clear separation in the coverage

score between the top scoring configuration and the next nearest neighbour, 3) relevance to the research question and 4) alignment with logic, theory and prior knowledge. Lastly, the identified candidate conditions were validated by investigating potential skewness in data and correlation between the candidate conditions.

Appendix 3

Solution visualization for positive outcome (having a successful collaboration with OHS within the preventive OHSM). The colours indicated workplaces covered by at least one of the solution pathways (SP) in the model where the outcome was present (Light grey = SP 1, grey = SP 2, dark grey = SP3. The grey colour indicates inconsistent workplaces identified by the model in which the outcome was not present. After excluding workplaces with missing data, this model included 109 of the 112 initial workplaces, hence. Note that the table is cropped.

No.	Workplace	OUTCOME	Close collaboration with OHS	Access to internal and external support in OHSM	Dialogue with OHS within OHSM	External demands for OHSM
1	161	1	1	1	0	1
2	70	1	1	1	0	1
3	79	1	1	1	0	0
4	108	1	1	1	0	1
5	146	1	1	1	0	1
6	164	1	1	0	0	1
7	179	1	1	0	0	1
8	133	1	1	1	0	1
9	14	1	1	1	1	0
10	109	1	1	1	1	0
11	176	1	1	1	1	0
12	82	1	1	1	1	1
13	118	1	1	1	1	1
14	72	1	1	1	1	1
15	126	1	1	1	1	1
16	13	1	1	1	1	1
17	25	1	1	1	1	1
18	29	1	1	1	1	1
19	46	1	1	1	1	1
20	48	1	1	0	1	1
21	16	1	1	0	1	1
22	137	1	1	0	1	1
23	24	1	0	0	1	1
24	36	1	0	0	1	1

25	119	1	0	0	1	1
26	52	1	0	1	1	1
27	65	1	0	1	1	1
28	186	1	0	1	1	1
29	33	1	0	1	1	1
30	22	1	0	1	1	0
31	34	1	0	1	1	0
32	63	1	0	1	1	1
33	125	1	0	1	1	1
34	135	1	0	1	1	1
35	31	1	0	1	1	0
36	174	1	0	1	1	1
37	81	1	0	1	0	1
38	50	1	0	0	0	1
39	84	1	0	1	0	1
40	127	1	0	1	0	0
41	166	1	0	1	0	0
42	113	1	0	1	0	1
43	169	1	0	1	0	1
44	105	1	0	0	0	1
45	107	1	0	1	0	1
46	112	1	0	0	0	1
47	136	1	0	0	0	0
48	159	1	0	1	0	1
49	20	1	0	1	0	1
50	32	1	0	1	0	1
51	44	1	0	0	0	0
52	35	0	1	0	0	1
53	18	0	1	1	1	0
54	77	0	0	1	1	0
55	59	0	0	1	1	0
56	60	0	0	1	1	1
57	99	0	0	0	1	1
58	102	0	0	0	1	1
59	40	0	0	1	0	1
60	145	0	0	1	0	1
.	.	.	.	.	.	.
.	.	.	.	.	.	.
109	154	0	0	1	0	1

Appendix 4.

Solution visualization for negative outcome (not having a successful collaboration with OHS within the preventive OHSM). The colours indicated workplaces covered by the solution pathway (SP) in the model where the outcome was present (green = SP 1). The grey colour indicates inconsistent workplaces identified by the model in which the outcome was not present. Note that the table is cropped.

No.	Workplace	OUTCOME	Dialogue with OHS within OHSM	Work environment challenge with physical strain
1	1	0	0	0
2	6	0	0	0
3	7	0	0	0
4	8	0	0	0
5	10	0	0	0
6	12	0	0	0
7	26	0	0	0
8	30	0	0	0
9	39	0	0	0
10	43	0	0	0
11	45	0	0	0
12	49	0	0	0
13	53	0	0	0
14	55	0	0	0
15	56	0	0	0
16	61	0	0	0
17	71	0	0	0
18	73	0	0	0
19	75	0	0	0
20	80	0	0	0
21	83	0	0	0
22	89	0	0	0
23	96	0	0	0
24	98	0	0	0
25	101	0	0	0
26	103	0	0	0
27	104	0	0	0
28	110	0	0	0
29	114	0	0	0
30	143	0	0	0
31	144	0	0	0
32	151	0	0	0
33	153	0	0	0
34	154	0	0	0
35	155	0	0	0
36	177	0	0	0
37	182	0	0	0
38	184	0	0	0
39	185	0	0	0
40	191	0	0	0

41	23	0	0	1
42	35	0	0	1
43	40	0	0	1
44	62	0	0	1
45	90	0	0	1
46	142	0	0	1
47	145	0	0	1
48	148	0	0	1
49	160	0	0	1
50	170	0	0	1
51	18	0	1	0
52	21	0	1	0
53	28	0	1	0
54	59	0	1	0
55	60	0	1	0
56	77	0	1	0
57	99	0	1	0
58	102	0	1	0
59	152	0	1	0
60	189	0	1	0
61	20	1	0	0
62	32	1	0	0
63	44	1	0	0
64	105	1	0	0
65	107	1	0	0
66	112	1	0	0
67	133	1	0	0
68	136	1	0	0
69	159	1	0	0
70	50	1	0	1
71	70	1	0	1
72	79	1	0	1
73	81	1	0	1
74	84	1	0	1
75	108	1	0	1
.	.	.	.	.
.	.	.	.	.
.	.	.	.	.
112	174	1	1	1
